# Effects of *Rhodiola rosea* on Physical and Decision-Making Performance in Football Players: A Randomised Controlled Trial

**DOI:** 10.3390/nu18050724

**Published:** 2026-02-24

**Authors:** Yue Dou, Yaqing Wang, Wei Zhang, Yuewei Jiang, Jiyao Zhang, Tao Yang, Ziqi Han, Yaotong Li, Chang Liu, Dingmeng Ren

**Affiliations:** 1 China Football College, Beijing Sport University, Beijing 100084, China; 2023210540@bsu.edu.cn (Y.D.);; 2 China Institute of Sport Science, General Administration of Sport of China, Beijing 100061, China; 3 School of Sport Science, Beijing Sport University, Beijing 100084, China

**Keywords:** sports nutrition, football players, *Rhodiola rosea* supplementation, fatigue resistance, performance maintenance under fatigue, high-intensity intermittent exercise, decision-making under fatigue

## Abstract

**Objectives**: To determine whether four weeks of *Rhodiola rosea* (RHO) supplementation improves intermittent exercise performance, post-exercise blood lactate concentrations, and decision-making under fatigue in competitive football players. **Methods:** Twenty-four male competitive football players completed a randomised, double-blind, placebo-controlled 4-week intervention (RHO vs. placebo). Outcomes included Yo-Yo IR2, repeated-sprint ability (RSA), post-RSA blood lactate (0, 3, 5 min), football-specific technical tests (passing and shooting), a video-based decision-making task (reaction time and accuracy), GPS-derived match running metrics, countermovement jump (CMJ), foot tapping (TAP), and haematological markers. **Results**: Yo-Yo IR2 performance significantly improved in the RHO group (*p* = 0.012) and was superior to the placebo group (*p* = 0.046). For RSA, mean sprint time improved significantly from pre- to post-intervention in the RHO group (*p* = 0.017), whereas no significant change was observed in the placebo group. Post-intervention, mean sprint time was significantly better in RHO than placebo (*p* = 0.041), with no between-group difference observed at baseline. Best sprint time showed no between-group difference (*p* = 0.723). Post-exercise blood lactate concentrations were significantly lower in RHO than placebo at 0, 3, and 5 min (all *p* < 0.05). Under fatigue, the RHO group demonstrated faster reaction time (*p* = 0.042) and higher decision accuracy (*p* = 0.049) than placebo. Additionally, the RHO group showed significant pre- to post-intervention improvements in passing and shooting performance (*p* < 0.05), with between-group differences observed only for short-pass performance. Match total and high-speed running distances were higher in RHO, accompanied by increases in haemoglobin and haematocrit (*p* < 0.05). **Conclusions:** Four weeks of *Rhodiola rosea* supplementation enhanced high-intensity intermittent performance and decision-making under fatigue, with findings suggesting improved performance maintenance rather than increased peak sprint capacity.

## 1. Introduction

In competitive sports research, sports nutrition supplements are widely used to support training adaptations, delay the development of fatigue, and maintain performance stability under high-intensity workloads [1]. Rather than focusing solely on enhancing single-bout maximal output, recent research has increasingly shifted attention toward the role of nutritional supplementation in supporting metabolic efficiency, recovery processes, and performance maintenance during repeated high-intensity loading, particularly when recovery windows are limited [2].

*Rhodiola rosea* (RHO) is a representative traditional herbal plant that has long been used across multiple regions to support physical activity and cope with stress-related conditions [3]. In particular, in high-altitude regions such as Tibet in China, *Rhodiola rosea* has been regarded as a traditional food-derived botanical and has been commonly incorporated into daily diets or used to support recovery following physical activity [4]. Building upon this long-standing experiential background, *Rhodiola rosea* has increasingly attracted attention in the fields of sports nutrition and exercise physiology in recent years. Previous studies indicate that, in the absence of evident adverse effects [5], RHO supplementation may exert beneficial effects on exercise performance and physiological adaptation, including attenuating post-exercise muscle discomfort and damage, enhancing antioxidant capacity, reducing oxidative stress, and lowering ratings of perceived exertion [6,7].

From a systems physiology perspective, RHO and its bioactive constituents have been reported to be involved in multiple regulatory pathways associated with exercise adaptation [8]. On the one hand, RHO is closely linked to mitochondrial energy metabolism regulatory networks, with its actions involving signalling axes such as AMP-activated protein kinase (AMPK), Sirt1, and PGC-1α [9,10], thereby supporting mitochondrial function and the efficiency of ATP resynthesis, which is of critical importance for energy recovery following high-intensity exercise [11]. On the other hand, RHO has also been reported to be associated with hypoxia-related signalling processes and regulatory pathways related to erythropoiesis, providing a physiological basis for its potential role in oxygen utilisation and recovery support [12]. In addition, some studies indicate that RHO may influence central nervous system stress responses and fatigue perception, thereby exerting coordinated effects at both peripheral metabolic and central regulatory levels [13].

Although existing studies have largely focused on endurance or hypoxia-based models to investigate the effects of *Rhodiola rosea* on energy metabolism and oxygen-transport-related physiological responses [14], its functional significance in high-intensity intermittent team-sport contexts remains insufficiently evaluated [15]. Moreover, findings regarding the ergogenic and metabolic effects of *Rhodiola rosea* supplementation remain inconsistent. Several studies have reported no significant improvements in exercise performance or metabolic responses following RHO supplementation, particularly when assessed under non-fatigued conditions, short intervention durations, or single-bout exercise protocols [16]. More specifically, direct evidence is still limited regarding whether RHO supplementation can produce consistent and functionally relevant effects across repeated high-intensity performance and fatigue-related functional outcomes during training or competition cycles. Moreover, existing research has predominantly concentrated on isolated fitness or physiological indicators, with relatively few studies integrating repeated high-intensity physical performance, neuromuscular function, haematological parameters, and more match-relevant technical and cognitive performance within a single research framework [17,18].

Football-specific performance relies on the capacity to sustain repeated high-intensity actions while preserving neuromuscular and cognitive function under fatigue, rather than on any single physical fitness attribute [19]. Football-specific actions impose substantial demands on lower-limb neuromuscular control and rapid energy system alternation, particularly when recovery between efforts is incomplete [20]. Under fatigued conditions, performance impairments extend beyond reductions in physical output and include decrements in movement control and decision-making consistency [21,22]. Accordingly, the present study focuses on performance maintenance under standardised fatigue conditions. Specifically, this study aimed to determine whether four weeks of *Rhodiola rosea* supplementation could enhance intermittent exercise capacity and decision-making performance under fatigue in competitive football players [23,24].

Based on these sport-specific performance demands, the present study constructed a testing framework centred on repeated high-intensity running capacity, neuromuscular control under limited recovery conditions, and the stability of technical execution and situational decision-making under fatigue [25]. Specifically, the Yo-Yo Intermittent Recovery Test Level 2 (Yo-Yo IR2) was employed to assess the ability to sustain high-intensity intermittent running under brief recovery conditions, in combination with GPS-derived indicators to characterise external load demands [26,27]. Furthermore, a repeated sprint ability (RSA) test was used as a standardised fatigue-induction task to elicit high-intensity intermittent fatigue under controlled conditions [28]. At the neuromuscular level, the countermovement jump (CMJ) and a rapid lower-limb repetitive movement test (foot tapping) were conducted under non-fatigued conditions [29,30]. On this basis, a situational decision-making test was administered following RSA-induced fatigue to evaluate decision reaction time and accuracy under a standardised fatigue background [31]. These outcomes were further integrated with sport-specific technical execution indicators to assess whether RHO supplementation could attenuate fatigue-induced declines in technical performance [32]. In addition, post-RSA blood lactate responses, together with haemoglobin and haematocrit indices, were monitored to provide physiological support for the observed performance changes [33].

Therefore, this study adopted a randomised, double-blind, placebo-controlled design to systematically investigate the effects of consecutive *Rhodiola rosea* supplementation on multidimensional performance outcomes in football players under high-intensity intermittent loading and fatigue conditions. These outcomes included high-intensity intermittent performance, neuromuscular function, physiological responses, and technical and decision-making performance under fatigue. We hypothesised that RHO supplementation would not primarily exert its effects by enhancing single-bout peak performance, but rather by improving energy metabolism and recovery efficiency, thereby enhancing performance maintenance capacity under repeated high-intensity loads and supporting technical execution and cognitive decision-making stability in fatigue-related match contexts, in closer alignment with the practical demands of football competition.

## 2. Materials and Methods

### 2.1. Study Design

**Figure 1:**
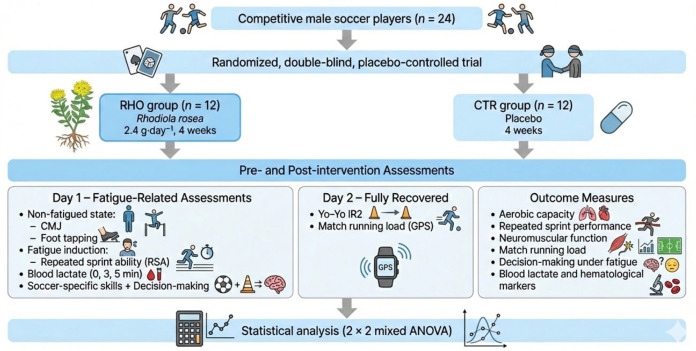
Diagram of the Experimental Procedure.

Using a randomised, double-blind, placebo-controlled experimental design, this study aimed to systematically investigate the effects of 4-week supplementation with a standardised *Rhodiola rosea* extract on high-intensity intermittent exercise performance and related physiological and biochemical indices in football players [34]. All tests were conducted before the start of the supplementation period (Pre, baseline) and within 24–48 h after completion of the intervention (Post).The overall study timeline and testing sequence are presented in Figure 1.

To minimise the confounding influence of acute fatigue on outcome measures across different performance domains, the testing sequence was deliberately structured according to fatigue status. Specifically, assessments were organised into three stages: (1) non-fatiguing baseline evaluations, (2) fatigue-inducing assessments and early recovery measurements, and (3) specialised performance assessments conducted after sufficient recovery. On day 1 of each testing cycle, following a standardised warm-up, participants first completed the countermovement jump (CMJ) and foot tapping (TAP) tests in a non-fatigued state to characterise baseline neuromuscular output. Subsequently, the repeated sprint ability (RSA) test was performed to induce short-term acute fatigue. Blood lactate samples were collected immediately after the RSA test and during the early recovery phase (0, 3, and 5 min) to characterise fatigue magnitude and early recovery kinetics. During the early recovery phase, when acute fatigue was still present, football-specific technical tests (short pass, long pass, and shooting) and situational decision-making tasks were performed sequentially to assess technical execution and cognitive performance under high-intensity loading conditions. On day 2 of each testing cycle, following a 24-h recovery period, the Yo-Yo Intermittent Recovery Test and external running load monitoring were conducted to evaluate specialised intermittent endurance capacity and match-related external load outputs, while minimising the residual effects of acute fatigue induced on day 1. The testing sequence was identical for both Pre and Post assessments.

### 2.2. Participants

**Figure 2:**
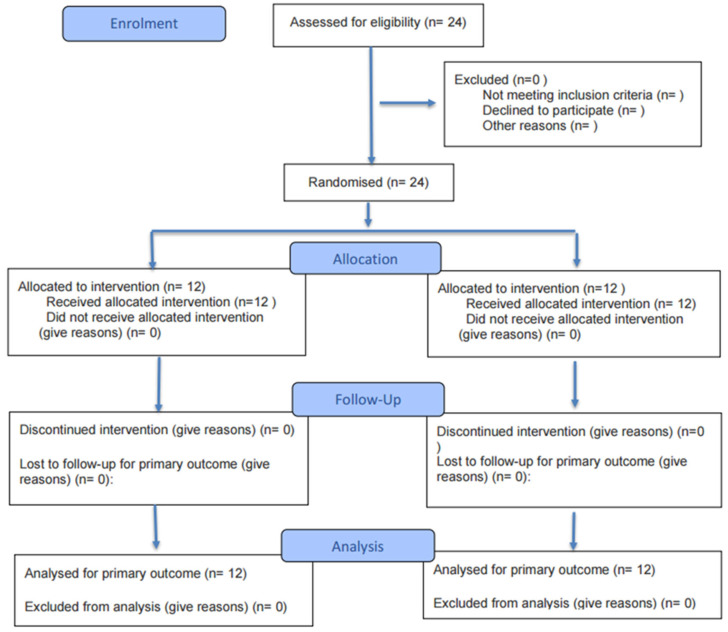
Flow diagram of the progress through the phases of a randomised trial of two groups (that is, enrolment, intervention allocation, follow-up, and data analysis).

Twenty-four male football players from Beijing Sport University were recruited (see Table 1 for participant characteristics). Inclusion criteria included a minimum of 3 years of systematic football training and stable training and competition status for at least 3 months preceding the study. Exclusion criteria included severe musculoskeletal injury or surgery within the previous 6 months, cardiovascular or metabolic disease, recent use of medications or supplements known to affect athletic performance or haematological indices, and known allergy to the ingredients of the study supplements. Participants were randomly allocated by a computer-generated randomisation sequence to either the *Rhodiola rosea* supplementation group (RHO) or the placebo control group (CTR), with 12 participants in each group (*n* = 12). The randomisation sequence was generated and concealed by researchers not involved in testing or statistical analysis. Participants, test administrators, and data analysts were blinded to group allocation until completion of the statistical analyses. The overall workflow of the process is summarized in Figure 2.

### 2.3. Ethical Review and Informed Consent

The study protocol was reviewed and approved by the Ethics Committee of Beijing Sport University (Approval No. 2025619H). All participants provided written informed consent after receiving a detailed explanation of the experimental procedures and potential risks. The trial was registered retrospectively at ClinicalTrials.gov (NCT07366320) on 16 January 2026, after the study had commenced, as registration was completed through an institution-wide centralized management process.

### 2.4. Intervention Protocol

The *Rhodiola rosea* supplement was purchased in capsule form from China Tongrentang Pharmaceutical Co., Ltd., Beijing, China.

According to the product specifications and study design, the daily dosage was set at 2.4 g. Salidroside was used as the marker compound, with a total daily content of 12 mg, and starch and gelatin (capsule shell) were used as excipients. To ensure consistency across the intervention period, all supplements were sourced from the same production batch and dispensed uniformly.

To standardise administration and maintain blinding, the contents of the RHO capsules were removed and prepared as a homogeneous suspension by an independent investigator not involved in testing or data analysis. This procedure was adopted to ensure identical appearance, taste, and administration between the RHO and placebo conditions. A placebo suspension with identical appearance, volume, and taste was prepared using the same procedure. Both solutions were dispensed in coded containers, and neither participants nor investigators were aware of group allocation throughout the study. The suspension was ingested orally once daily at a consistent time of day. Supplement administration was performed by personnel not involved in testing or data analysis. Supplement compliance was monitored throughout the intervention period.

Participants in the RHO group ingested the supplement daily for 4 consecutive weeks according to the established protocol, while the control group consumed a placebo solution with identical appearance, volume, and taste at the same time points. Throughout the study period, all participants were instructed to maintain their habitual training schedules and dietary routines and to refrain from using any additional nutritional supplements or medications that could influence exercise performance.The daily dietary nutrient composition of participants during the study period is presented in Table 2.

### 2.5. Countermovement Jump (CMJ)

**Figure 3:**
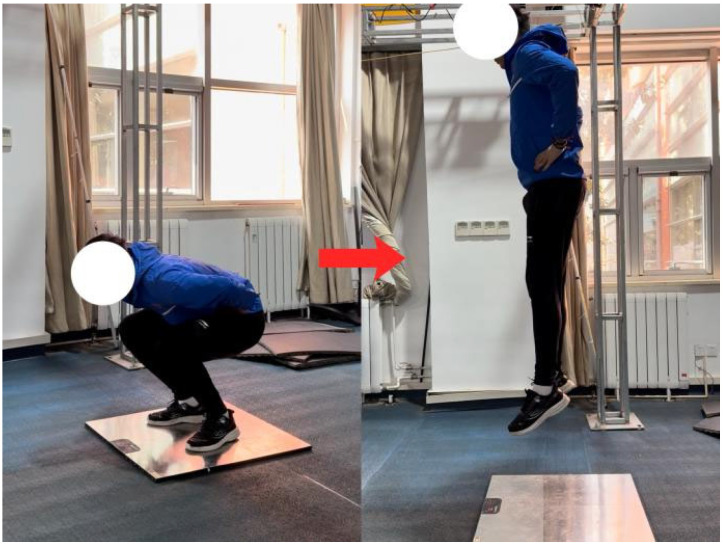
Countermovement Jump.

Countermovement jump (CMJ) performance was assessed using a force platform. Participants performed the jump with hands placed on the hips, starting from an upright standing position, followed by a rapid downward movement and an immediate vertical jump. Each participant completed three trials, with a rest interval of 60 s between trials. The best trial was retained for analysis. The CMJ was administered following a standardised warm-up and prior to the foot tapping (TAP) test.The CMJ testing procedure is illustrated in Figure 3. 

### 2.6. Foot Tapping

**Figure 4:**
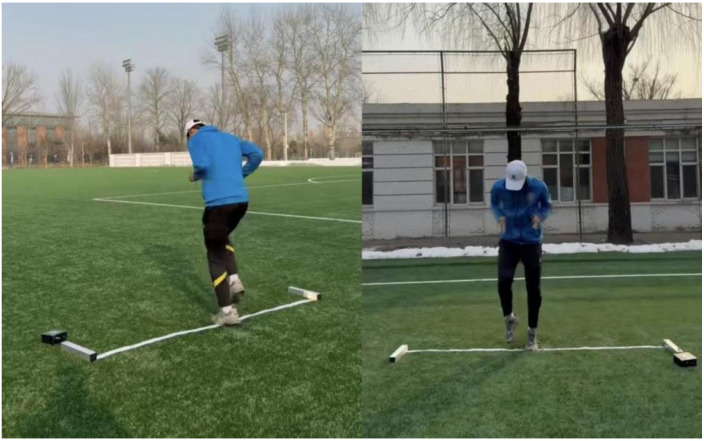
Foot Tapping Count.

The foot tapping (TAP) test was administered on a flat, clear surface to assess lower-limb movement frequency. Participants assumed a starting position with both feet on a designated mark. Following a standardized command, they executed continuous, rapid foot taps aiming to maximize the number of valid contacts within a set time period, with minimal ground contact time and without forceful stomping. They were instructed to avoid any clear jumping motion or substantial elevation of the feet from the surface [29]. The TAP test was administered following the countermovement jump (CMJ) test and prior to the repeated sprint ability (RSA) test. The TAP test setup and procedure are illustrated in Figure 4.

### 2.7. Repeated Sprint Ability Test (RSA)

**Figure 5:**
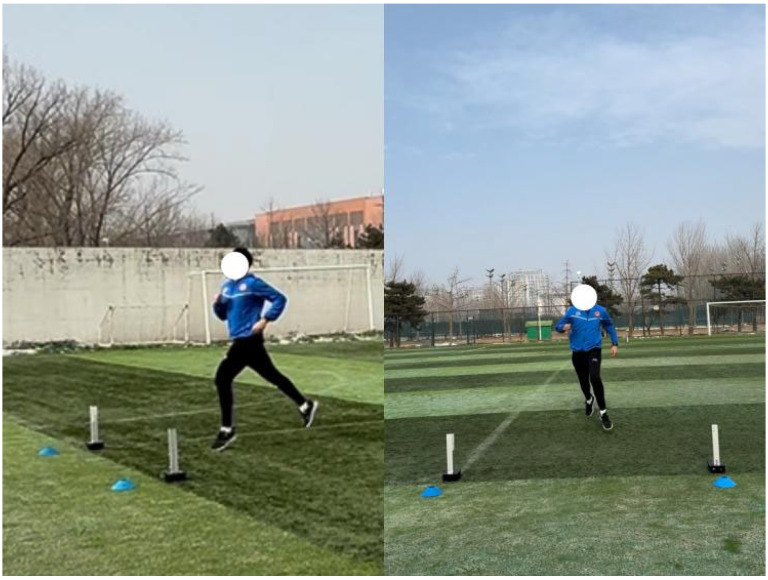
Repeated Sprint Ability.

On a flat football field, a 7 × 30 m repeated sprint protocol with 25 s of passive recovery between sprints was employed. Sprints were initiated from a stationary start, and sprint times were recorded using electronic timing gates. The RSA best and RSA mean sprint times were recorded as the primary performance metrics [35].The RSA testing protocol is illustrated in Figure 5.

### 2.8. Short Passing Ability Test

**Figure 6:**
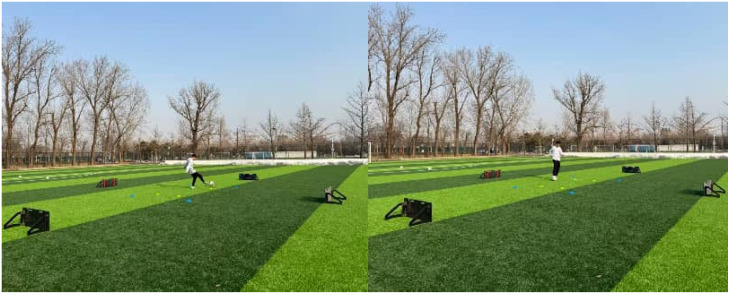
Loughborough Soccer Passing Test.

The Loughborough Soccer Passing Test (LSPT) was conducted within a rectangular area measuring 12 × 9 m, with rebound boards positioned along all four sides to serve as passing targets. Participants completed a sequence of short passes in a predetermined order, aiming to perform the task as quickly and accurately as possible. Penalty time was uniformly applied for errors, including missed targets, incorrect target selection, ball control errors, or rule violations. Test performance was quantified as the total time, calculated as the sum of completion time and accumulated penalty time, with shorter total times indicating better short-passing performance. The short-passing test was administered post-RSA during early recovery to assess passing stability under time pressure in a fatigued state, with identical Pre and Post testing sequences. The short-passing test setup is illustrated in Figure 6.

### 2.9. Long Passing Ability Test

**Figure 7:**
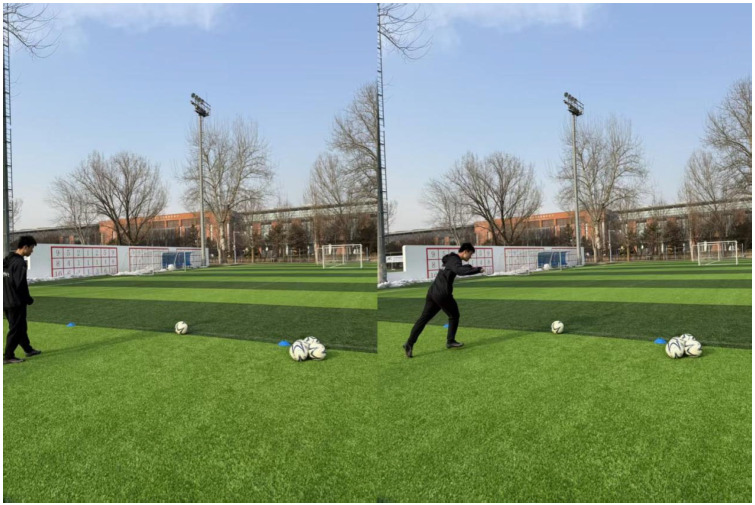
Long-Distance Passing Ability Test.

A standardized long-range passing test was administered on a level playing field. A standard five-a-side goal served as the target area, with passes delivered from a stationary point 28 m away. Each participant executed six consecutive long passes with the instruction to project the ball into the goal area as accurately as possible. The landing position of each pass was determined by its first bounce, and passes whose first bounce landed within the goal area were deemed successful. This test primarily assessed long-pass accuracy rather than maximal kicking distance. The long-passing test setup is illustrated in Figure 7.

### 2.10. Shooting Proficiency Test

**Figure 8:**
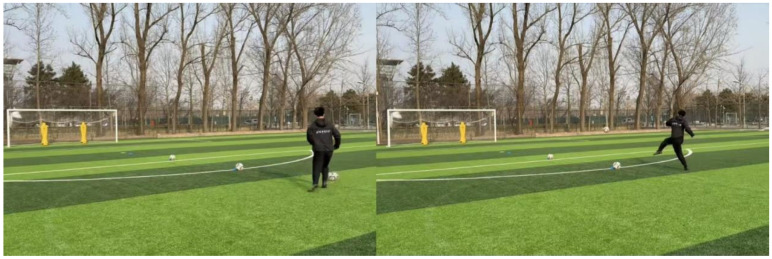
Loughborough Soccer Shooting Test.

The shooting test was performed using a standard 11-a-side football goal, with two inflatable dummies positioned on the goal line to simulate defensive interference. Fixed shooting positions were established directly in front of the goal at distances of 12, 16, and 20 m from the goal line, representing close-, medium-, and long-range shooting tasks, respectively. Participants executed one shot from each of the three prescribed positions, resulting in a total of three shots. No restrictions were imposed on the preferred foot or shooting technique. Participants were instructed to perform each shot as accurately as possible while adhering to standard technical requirements. Shooting performance was scored according to the Loughborough Soccer Shooting Test (LSST) criteria [36]. Points were awarded based on the target zone in which the ball first landed, and the total score was calculated as the sum of points across all shots. Higher total scores indicated better shooting accuracy and technical execution consistency. The shooting test setup and scoring zones are illustrated in Figure 8.

### 2.11. Football Decision-Making Test

Football-specific decision-making ability was assessed using a reaction time task based on real match video scenarios. Test stimuli consisted of attack-related video clips (4–8 s), in which key decision moments were temporally occluded to prevent responses based on subsequent play outcomes. Similar video-based decision-making paradigms have been widely used in previous football research to evaluate decision reaction time and accuracy under sport-specific perceptual conditions [37].

**Figure 9:**
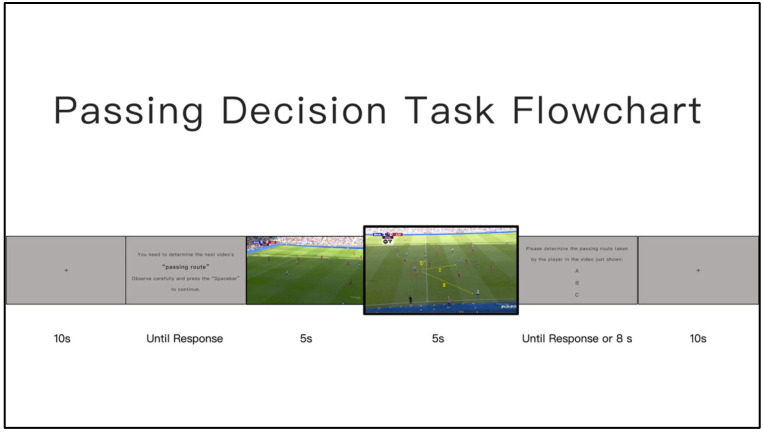
Decision-Making Ability.

Testing was conducted in a quiet indoor environment, and participants were instructed to respond as quickly and accurately as possible via keyboard input. The experimental program automatically recorded reaction times and response accuracy. Response accuracy was defined as agreement with the expert-defined optimal decision for each scenario. Prior to the formal test, participants completed six familiarisation trials. The formal test consisted of 30 video trials presented in a randomised order. Decision reaction time (RT) was defined as the time interval (ms) from the onset of visual masking to the first valid response. The decision-making task procedure is illustrated in Figure 9. 

### 2.12. Yo-Yo IR2 Intermittent Recovery Test

**Figure 10:**
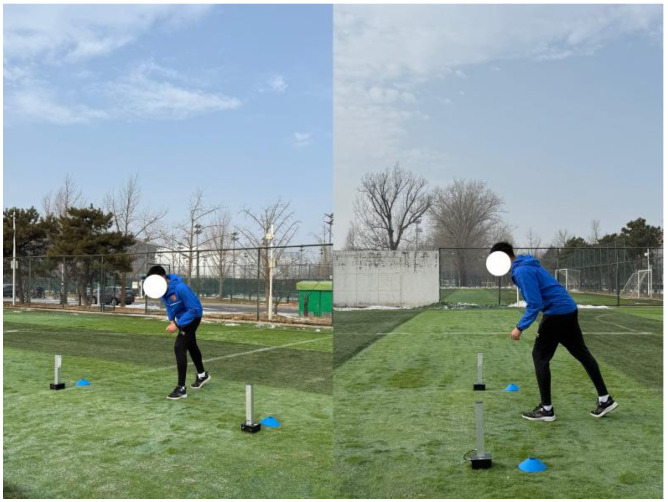
Yo-Yo Intermittent Recovery Test Level 2.

The Yo-Yo Intermittent Recovery Test Level 2 (Yo-Yo IR2) protocol required participants to perform repeated 20 m shuttle runs in response to auditory signals, with running speed progressively increasing. Each shuttle run was followed by a 10 s active recovery period involving jogging, and the test continued until volitional exhaustion. Total distance covered (m) was recorded as the primary outcome measure [38]. The Yo-Yo IR2 protocol is illustrated in Figure 10. 

### 2.13. Match Running Load Monitoring

**Figure 11:**
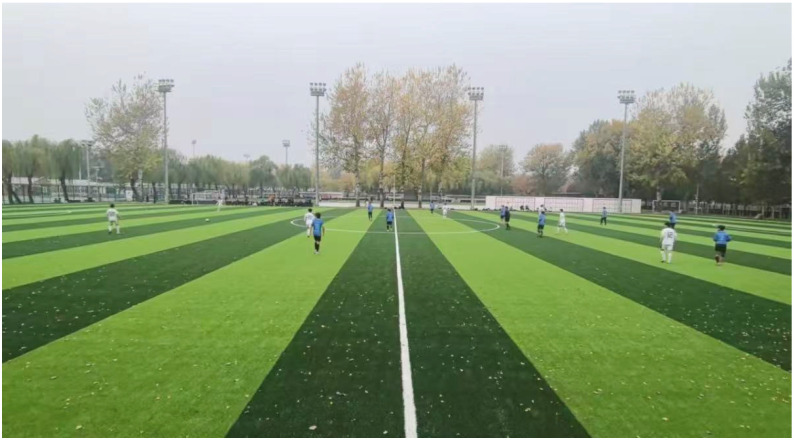
Match Running Load Monitoring.

To monitor external running loads during match play, participants wore Catapult X7 GPS devices (Catapult Sports, Melbourne, Australia) to collect match-running data [39]. The following variables were recorded: total distance covered, high-speed running distance (20–25 km·h^−1^), sprint distance (≥25 km·h^−1^), acceleration count (≥3 m·s^−2^), and deceleration count (≤−3 m·s^−2^) [40,41]. All data were collected during formal 11-a-side matches in which the participants competed. Match conditions, including opponents and tactical formations, were kept as consistent as possible between the Pre and Post assessments. All participants wore the same GPS device model during each match. Following each match, raw data were downloaded and processed using the Catapult One: Sports Training application on an iPad (iPadOS; Catapult Sports, Melbourne, Australia). Data screening, quality control, and final export were performed by a single researcher to ensure consistency across data processing. To minimise the potential influence of acute fatigue on external load characterisation, both Pre and Post match monitoring sessions were scheduled within the Day 2 testing window. The match running load monitoring procedure is illustrated in Figure 11. 

### 2.14. Blood Parameter Tests (BLa, Hb/Hct)

Blood lactate concentration (BLa) was measured using a portable lactate analyser (LactateScout, H/P COSMOS, Nussdorf-Traunstein, Germany) [42]. Approximately 5 μL of capillary blood was obtained from the fingertip: the puncture site was disinfected with alcohol and allowed to dry, a single-use lancet was used for sampling, the first drop of blood was discarded, and the second drop was applied to the test strip for analysis. Measurements were taken immediately after the RSA test (0 min), and at 3 min and 5 min post-exercise.

Haemoglobin (Hb) concentration and haematocrit (Hct) were assessed at baseline and after the intervention. Venous blood samples (2 mL) were collected from the antecubital vein into EDTA tubes in the early morning under fasting conditions, following at least 10 min of seated rest. All participants fasted for 8–12 h prior to blood collection. Sampling was conducted between 08:00 and 08:30. Hb and Hct were analysed using a fully automated haematology analyser.

### 2.15. Test–Retest Reliability

Test–retest reliability, assessed during the familiarisation sessions, indicated acceptable to high consistency across all fitness, technical, and decision-making tests (intraclass correlation coefficient (ICC) range: 0.90–0.95; coefficient of variation (CV) range: 5–9%).

### 2.16. Statistical Analysis

Statistical analyses were performed using SPSS software (version 26.0; IBM Corp., Armonk, NY, USA). All continuous variables are presented as mean ± standard deviation (SD). Data normality was assessed using the Shapiro–Wilk test, and homogeneity of variance was examined using Levene’s test. When the assumptions were satisfied, parametric statistical procedures were applied.

Group- and time-related effects were examined using an ordinary two-way analysis of variance (ANOVA) with group (RHO vs. CTR) and time (Pre vs. Post) as fixed factors. When appropriate, post hoc comparisons were conducted using Fisher’s least significant difference (LSD) test to explore between-group and within-group differences.

To quantify the magnitude of observed differences, effect sizes were calculated as Hedges’ g, with corresponding 95% confidence intervals (CI). Effect sizes were interpreted as small (0.2), moderate (0.5), or large (0.8). The calculation of effect sizes was based on the pooled standard deviation derived from the residual mean square of the ANOVA model, with small-sample correction applied.

An a priori sample size estimation was conducted using G*Power software (version 3.1.9.7). Based on an expected moderate effect size (f = 0.25), an alpha level of 0.05, and a statistical power of 0.80 for a two-factor ANOVA design, a minimum total sample size of 24 participants was required. This requirement was met in the present study.

All statistical tests were performed with a significance level set at *p* < 0.05.

## 3. Results

### 3.1. Aerobic Capacity and Repeated Sprint Performance

**Figure 12:**
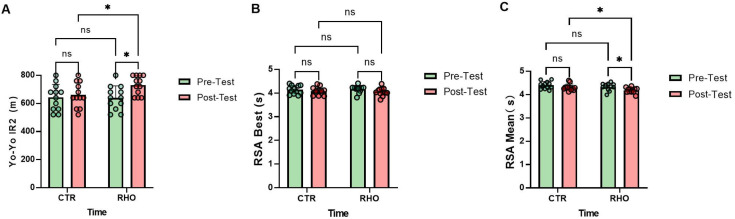
Aerobic Capacity and Repeated Sprint Performance. (**A**) Yo-Yo IR2 (m); (**B**) RSA Best (s); (**C**) RSA Mean (s). Dots represent individual participants. * *p* < 0.05; ns, not significant.

As illustrated in Figure 12, no significant differences were observed between groups at baseline in the Yo-Yo IR2 test. Compared with baseline, the *Rhodiola rosea* group (RHO) showed a significant increase in Yo-Yo IR2 running distance after the intervention (*p* = 0.012, Hedges’ g = 1.04, 95% CI for the mean change: [21.22, 158.8] m), whereas no significant change was observed in the placebo control group (CTR) from pre- to post-intervention (*p* = 0.628). In the post-intervention between-group comparison, the RHO group achieved a significantly greater Yo-Yo IR2 distance than the CTR group (*p* = 0.046, Hedges’ g = 0.82, 95% CI for the between-group mean difference: [1.22, 138.8] m), whereas no significant difference was detected between groups at baseline (*p* = 0.923; Figure 12A).

The RHO group showed no significant change in RSA best from pre- to post-intervention (*p* = 0.114), and the CTR group likewise exhibited no significant change (*p* = 0.393). In the post-intervention between-group comparison, no significant difference was observed in RSA best between the RHO and CTR groups (*p* = 0.723), and similarly, no significant difference was present at baseline (*p* = 0.695; Figure 12B).

The RHO group demonstrated a significant improvement in RSA mean from pre- to post-intervention (*p* = 0.017, Hedges’ g = 0.98, 95% CI for the mean change: [0.0266, 0.2601] s), whereas no significant change was observed in the CTR group (*p* = 0.108). In the post-intervention between-group comparison, the RHO group exhibited a significantly better RSA mean than the CTR group (*p* = 0.041, Hedges’ g = 0.83, 95% CI for the between-group mean difference: [0.00493, 0.2384] s), whereas no significant between-group difference was observed at baseline (*p* = 0.212; Figure 12C).

### 3.2. Neuromuscular Performance

**Figure 13:**
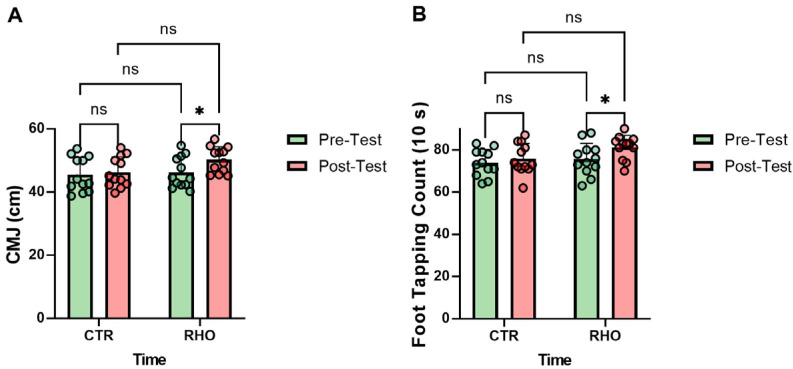
Neuromuscular Performance. (**A**) Countermovement Jump (cm); (**B**) Foot Tapping Count (10 s). Dots represent individual participants. * *p* < 0.05; ns, not significant.

As illustrated in Figure 13, the *Rhodiola rosea* group (RHO) showed a significant increase in countermovement jump (CMJ) performance from pre- to post-intervention (*p* = 0.046, Hedges’ g = 0.81, 95% CI for the mean change: [0.07, 8.04] cm), whereas no significant change was observed in the placebo control group (CTR) (*p* = 0.709). In the post-intervention between-group comparison, the difference in CMJ performance between the RHO and CTR groups did not reach statistical significance (*p* = 0.052), and no significant between-group difference was observed at baseline (*p* = 0.753; Figure 13A).

The RHO group showed a significant increase in foot-tapping frequency from pre- to post-intervention (*p* = 0.046, Hedges’ g = 0.84, 95% CI for the mean change: [0.12, 11.22] counts (10 s)), whereas no significant change was observed in the CTR group (*p* = 0.490). In the post-intervention between-group comparison, the difference in foot-tapping frequency between the RHO and CTR groups did not reach statistical significance (*p* = 0.056), and no significant between-group difference was observed at baseline (*p* = 0.548; Figure 13B).

### 3.3. External Load Indicators in Match Conditions

**Figure 14:**
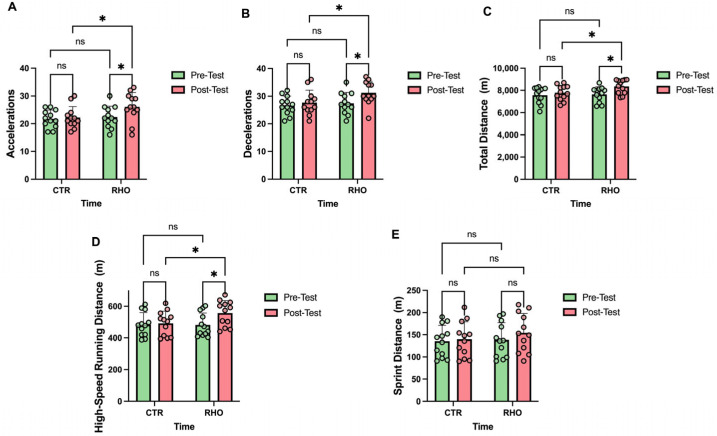
Match-Derived External Load Variables. (**A**) Accelerations (count); (**B**) Decelerations (count); (**C**) Total distance covered (m); (**D**) High-speed running distance (m); (**E**) Sprint distance (m). Dots represent individual participants. * *p* < 0.05; ns, not significant.

Changes in match running load indicators are presented in Figure 14, the RHO group showed a significant increase in acceleration counts from pre- to post-intervention (*p* = 0.033, Hedges’ g = 0.87, 95% CI for the mean change: [0.31, 7.03] counts), whereas no significant change was observed in the CTR group (*p* = 0.766). In the post-intervention between-group comparison, the RHO group exhibited a significantly higher number of accelerations than the CTR group (*p* = 0.026, Hedges’ g = 0.91, 95% CI for the between-group mean difference: [0.47, 7.20] counts), whereas no significant between-group difference was observed at baseline (*p* = 0.691; Figure 14A).

The RHO group showed a significant increase in deceleration counts from pre- to post-intervention (*p* = 0.027, Hedges’ g = 0.90, 95% CI for the mean change: [0.45, 7.05] counts), whereas no significant change was observed in the CTR group (*p* = 0.544). In the post-intervention between-group comparison, the RHO group exhibited a significantly higher number of decelerations than the CTR group (*p* = 0.038, Hedges’ g = 0.84, 95% CI for the between-group mean difference: [0.20, 6.80] counts), whereas no significant between-group difference was observed at baseline (*p* = 0.649; Figure 14B).

The RHO group showed a significant increase in total running distance from pre- to post-intervention (*p* = 0.014, Hedges’ g = 1.01, 95% CI for the mean change: [147.6, 1252] m), whereas no significant change was observed in the CTR group (*p* = 0.460). In the post-intervention between-group comparison, the RHO group exhibited a significantly greater total running distance than the CTR group (*p* = 0.044, Hedges’ g = 0.82, 95% CI for the between-group mean difference: [15.99, 1121] m), whereas no significant between-group difference was observed at baseline (*p* = 0.792; Figure 14C).

The RHO group showed a significant increase in high-speed running distance from pre- to post-intervention (*p* = 0.022, Hedges’ g = 0.94, 95% CI for the mean change: [11.04, 137.5] m), whereas no significant change was observed in the CTR group (*p* = 0.790). In the post-intervention between-group comparison, the RHO group exhibited a significantly greater high-speed running distance than the CTR group (*p* = 0.046, Hedges’ g = 0.81, 95% CI for the between-group mean difference: [1.12, 127.5] m), whereas no significant between-group difference was observed at baseline (*p* = 0.962; Figure 14D).

The RHO group showed no significant change in sprint distance from pre- to post-intervention (*p* = 0.333), and the CTR group likewise exhibited no significant change (*p* = 0.798). In the between-group comparison, no significant difference was observed between the two groups at baseline (*p* = 0.862), and sprint distance also did not differ significantly between groups after the intervention (*p* = 0.374; Figure 14E).

### 3.4. Football-Specific Technical Performance

**Figure 15:**
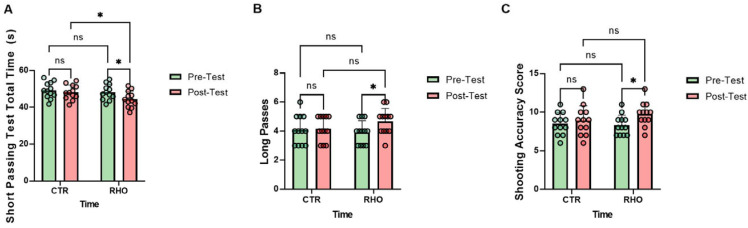
Football-Specific Technical Performance Variables. (**A**) Short passing test total time (s); (**B**) Number of successful long passes (count); (**C**) Shooting accuracy score. Dots represent individual participants. * *p* < 0.05; ns, not significant.

As illustrated in Figure 15, the RHO group showed a significant reduction in total completion time for the short-passing test from pre- to post-intervention (*p* = 0.033, Hedges’ g = 0.87, 95% CI for the mean change: [0.329, 7.321] s), whereas no significant change was observed in the CTR group (*p* = 0.540). In the post-intervention between-group comparison, the RHO group exhibited a significantly shorter total completion time than the CTR group (*p* = 0.036, Hedges’ g = 0.85, 95% CI for the between-group mean difference: [0.266, 7.257] s), whereas no significant between-group difference was observed at baseline (*p* = 0.564; Figure 15A).

The RHO group showed a significant improvement in long-pass performance from pre- to post-intervention (*p* = 0.043, Hedges’ g = 0.82, 95% CI for the mean change: [0.02, 1.48] points), whereas no significant change was observed in the CTR group (*p* = 0.818). In the post-intervention between-group comparison, the difference in long-pass performance between the RHO and CTR groups did not reach statistical significance (*p* = 0.172), and no significant between-group difference was observed at baseline (*p* = 0.645; Figure 15B).

The RHO group showed a significant increase in shooting accuracy scores from pre- to post-intervention (*p* = 0.033, Hedges’ g = 0.87, 95% CI for the mean change: [0.12, 2.71] points), whereas no significant change was observed in the CTR group (*p* = 0.520). In the post-intervention between-group comparison, the difference in shooting accuracy scores between the RHO and CTR groups did not reach statistical significance (*p* = 0.201, Hedges’ g = 0.51, 95% CI [0.46, 2.13]), and no significant between-group difference was observed at baseline (*p* = 0.796; Figure 15C).

### 3.5. Decision-Making Performance

**Figure 16:**
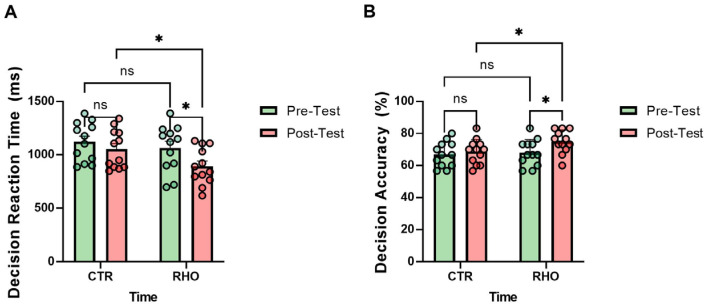
Decision-Making Performance. (**A**) Decision Reaction Time (ms); (**B**) Decision Accuracy (%). Dots represent individual participants. * *p* < 0.05; ns, not significant.

As illustrated in Figure 16, the RHO group showed a significant reduction in decision reaction time from pre- to post-intervention (*p* = 0.030, Hedges’ g = 0.86, 95% CI for the mean change: [17.24, 326.6] ms), whereas no significant change was observed in the CTR group (*p* = 0.376). In the post-intervention between-group comparison, the RHO group exhibited significantly faster decision reaction times than the CTR group (*p* = 0.042, Hedges’ g = 0.83, 95% CI for the between-group mean difference: [6.25, 315.6] ms), whereas no significant between-group difference was observed at baseline (*p* = 0.457; Figure 16A).

The RHO group showed a significant increase in decision accuracy from pre- to post-intervention (*p* = 0.034, Hedges’ g = 0.87, 95% CI for the mean change: [0.57, 13.31] percentage points), whereas no significant change was observed in the CTR group (*p* = 0.602). In the post-intervention between-group comparison, the RHO group exhibited significantly higher decision accuracy than the CTR group (*p* = 0.049, Hedges’ g = 0.80, 95% CI for the between-group mean difference: [0.02, 12.76] percentage points), whereas no significant between-group difference was observed at baseline (*p* = 0.727; Figure 16B).

### 3.6. Blood Lactate and Haematological Parameters

**Figure 17:**
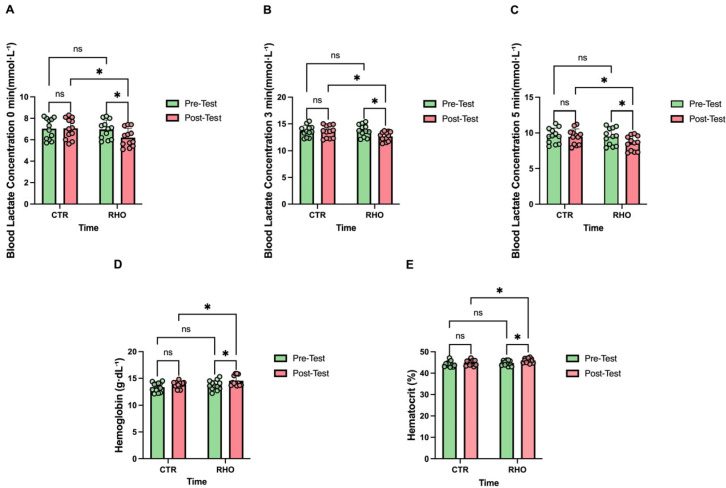
Blood Lactate Responses and Hematological Parameters. (**A**) Blood lactate concentration at 0 min post-exercise (mmol·L^−1^); (**B**) Blood lactate concentration at 3 min post-exercise (mmol·L^−1^); (**C**) Blood lactate concentration at 5 min post-exercise (mmol·L^−1^); (**D**) Hemoglobin concentration (g·dL^−1^); (**E**) Hematocrit (%). Dots represent individual participants. * *p* < 0.05; ns, not significant.

Changes in blood lactate responses and haematological parameters are presented in Figure 17. Compared with baseline, the RHO group showed a significant reduction in 0-min blood lactate concentration after the intervention (*p* = 0.046, Hedges’ g = 0.82, 95% CI for the mean change: [0.01, 1.52] mmol·L^−1^), whereas no significant change was observed in the CTR group from pre- to post-intervention (*p* = 0.929). In the post-intervention between-group comparison, the RHO group exhibited a significantly lower 0-min blood lactate concentration than the CTR group (*p* = 0.028, Hedges’ g = 0.91, 95% CI for the between-group mean difference: [0.10, 1.60] mmol·L^−1^), whereas no significant difference was detected between groups at baseline (*p* = 0.894; Figure 17A).

Compared with baseline, the RHO group showed a significant reduction in 3-min blood lactate concentration after the intervention (*p* = 0.016, Hedges’ g = 1.01, 95% CI for the mean change: [0.21, 1.93] mmol·L^−1^), whereas no significant change was observed in the CTR group from pre- to post-intervention (*p* = 0.816). In the post-intervention between-group comparison, the RHO group exhibited a significantly lower 3-min blood lactate concentration than the CTR group (*p* = 0.046, Hedges’ g = 0.82, 95% CI for the between-group mean difference: [0.02, 1.73] mmol·L^−1^), whereas no significant difference was detected between groups at baseline (*p* = 0.831; Figure 17B).

Compared with baseline, the RHO group showed a significant reduction in 5-min blood lactate concentration after the intervention (*p* = 0.039, Hedges’ g = 0.85, 95% CI for the mean change: [0.05, 1.84] mmol·L^−1^), whereas no significant change was observed in the CTR group from pre- to post-intervention (*p* = 0.751). In the post-intervention between-group comparison, the RHO group exhibited a significantly lower 5-min blood lactate concentration than the CTR group (*p* = 0.049, Hedges’ g = 0.81, 95% CI for the between-group mean difference: [0.01, 1.79] mmol·L^−1^), whereas no significant difference was detected between groups at baseline (*p* = 0.823; Figure 17C).

Compared with baseline, the RHO group showed a significant increase in haemoglobin (Hb) after the intervention (*p* = 0.0216, Hedges’ g = 0.96, 95% CI for the mean change: [0.126, 1.508] g·dL^−1^), whereas no significant change was observed in the CTR group from pre- to post-intervention (*p* = 0.196). In the post-intervention between-group comparison, the RHO group exhibited significantly higher Hb than the CTR group (*p* = 0.0424, Hedges’ g = 0.84, 95% CI for the between-group mean difference: [0.0258, 1.408] g·dL^−1^), whereas no significant difference was detected between groups at baseline (*p* = 0.313; Figure 17D).

Compared with baseline, the RHO group showed a significant increase in haematocrit after the intervention (*p* = 0.0125, Hedges’ g = 1.05, 95% CI for the mean change: [0.28, 2.20] percentage points), whereas no significant change was observed in the CTR group from pre- to post-intervention (*p* = 0.234). In the post-intervention between-group comparison, the RHO group exhibited a significantly higher haematocrit than the CTR group (*p* = 0.033, Hedges’ g = 0.88, 95% CI for the between-group mean difference: [0.09, 2.01] percentage points), whereas no significant difference was detected between groups at baseline (*p* = 0.426; Figure 17E).

## 4. Discussion

The results of this study indicate that after four consecutive weeks of *Rhodiola rosea* supplementation, football players exhibited significant improvements in Yo-Yo IR2 performance and RSA mean sprint time, whereas RSA best sprint time remained unchanged. Concurrently, CMJ height and foot tapping (TAP) frequency increased; acceleration and deceleration counts during match scenarios rose, while sprint distance remained stable; decision-making reaction time shortened with improved accuracy; blood lactate concentrations at 0, 3, and 5 min post-exercise decreased significantly; and haemoglobin and haematocrit levels increased following the intervention [43,44].

The improvement in Yo-Yo IR2 performance reflects enhanced recovery and reactivation capacity during high-intensity intermittent running. This test correlates not only with maximal aerobic capacity but also depends heavily on the rate of energy resynthesis and the restoration of metabolic homeostasis within short recovery windows [28]. RSA results further delineate the mechanism of action: while RSA mean improved, RSA best remained unchanged, suggesting that *Rhodiola rosea* positively influences speed maintenance and fatigue tolerance during repeated sprint efforts [45]. *Rhodiola rosea* likely acts on metabolic recovery, acid-base/ion homeostasis, and output stability during short recovery periods, thereby enhancing speed maintenance capacity during repeated sprints [46]. Optimal sprint time, however, more closely approximates single-peak neuromuscular explosive capacity, primarily determined by maximum strength, maximum power, and instantaneous neural drive levels [47]. Existing evidence indicates that *Rhodiola rosea* exerts anti-fatigue effects rather than directly promoting peak output, consistent with the findings of this study [48,49].

Under match conditions, the significant increase in acceleration and deceleration events without corresponding changes in sprint distance further substantiates *Rhodiola rosea*’s specific support for high-frequency stop–start and variable-speed actions. Acceleration and deceleration represent actions demanding greater energy expenditure and neuromuscular cost in football, relying on rapid eccentric-to-concentric transitions and energy resupply during brief recovery intervals [50,51]. This performance characteristic logically aligns with improvements in average RSA scores and lactate kinetics, underscoring *Rhodiola rosea*’s role in supporting the repeatability of high-intensity actions during matches [52,53]. Following repeated sprint loads, the *Rhodiola rosea* group demonstrated higher execution efficiency and accuracy in football-specific technical tests, including short passes, long passes, and shooting. The findings suggest that enhanced fatigue tolerance and energy resupply capacity may enable athletes to maintain fine motor control under high-load conditions [23].

In terms of cognitive performance, *Rhodiola rosea* supplementation significantly improved decision-making reaction time and accuracy following fatigue [43]. Previous studies suggest that high-intensity exercise-induced metabolic disruption and subjective fatigue impair attentional allocation and delay information processing [43]. *Rhodiola rosea* may alleviate central metabolic and stress burdens under high-load conditions by reducing peripheral fatigue and stress responses, thereby helping to maintain the stability of executive control and decision-making processes [54]. These findings corroborate the improvements observed in football-specific technical tests. Furthermore, research indicates that *Rhodiola rosea* may exert indirect cognitive support by modulating stress responses and fatigue perception, aligning with the observed synergistic improvement in physical fitness, metabolism, and cognition [55]. On this basis, the integration of GPS-derived match metrics with these standardized functional assessments provides a dual-layer validation of *Rhodiola rosea*’s efficacy [56]. While match fatigue is multifactorial, involving energy depletion, muscle damage, and central fatigue, the significant increase in acceleration and deceleration counts suggests that the metabolic and neuromuscular benefits observed in the laboratory translate into enhanced work rate during real competition [57]. By utilizing a standardized RSA-induced fatigue profile to isolate the “performance maintenance” signal from match-play noise, this study demonstrates that *Rhodiola rosea*’s support for lactate kinetics and decision-making stability is consistent across varying peak-intensity scenarios. This robust evidence chain supports *Rhodiola rosea* as a viable ergogenic aid for maintaining both physical and cognitive integrity under match-related constraints [58].

Blood lactate levels decreased significantly at 0, 3, and 5 min post-exercise, providing direct physiological evidence for accelerated recovery kinetics. This reduction in blood lactate likely reflects enhanced lactate clearance and oxidative reutilisation, rather than diminished lactate production. This, in turn, accelerates phosphagen resynthesis and restores internal homeostasis [59], supporting the concurrent improvement in Yo-Yo IR2 and RSA average scores. Concurrently, elevations in Hb and Hct may theoretically enhance blood oxygen-carrying capacity, potentially supporting oxygen supply and lactate oxidation during recovery [60]. However, Hb/Hct ratios are susceptible to variations in plasma volume, and as this study did not perform plasma volume correction or directly measure Hb mass, these findings are more appropriately interpreted as ‘haematological alterations related to oxygen transport’ rather than directly equated with haematopoietic adaptation [61]. Subsequent studies should incorporate plasma volume correction, Hb mass, reticulocyte count, and haematopoietic regulatory markers to clarify their physiological origins and relationship with enhanced athletic performance [62].

*Rhodiola rosea* may improve intermittent running capacity and recovery-related outcomes through complementary peripheral mechanisms at the skeletal muscle level; the selective improvement in performance maintenance and the faster post-exercise lactate decline is consistent with enhanced oxidative recovery processes within myocytes, including more efficient lactate clearance and oxidative reutilisation during early recovery, which would support repeated high-intensity efforts and phosphagen resynthesis [63]. In parallel, oxygen transport-related pathways may also contribute [15]. *Rhodiola rosea* has been reported to interact with hypoxia-responsive signalling, and a plausible route involves modulation of erythropoietin regulation in renal and hepatic cells, with downstream actions on erythroid progenitor cells in the bone marrow that influence erythrocyte-related indices and oxygen delivery capacity [63,64]. However, the present study did not assess molecular mediators such as HIF-1α or erythropoietin, and the observed haemoglobin and haematocrit changes should be interpreted cautiously because plasma volume shifts cannot be excluded [65]. Collectively, these mechanisms remain interpretative, and future studies should incorporate targeted molecular and cellular measurements together with plasma volume correction and indices of erythropoiesis and muscle oxidative function to verify the proposed pathways.

The concurrent enhancement of CMJ height and foot tapping performance (TAP) in this experiment suggests an improvement in the fundamental functional state of athletes’ neuromuscular output. The CMJ is frequently employed to characterise athletes’ neuromuscular status, whilst TAP has also been demonstrated to effectively evaluate lower-limb neuromuscular capacity in footballers [29]. In this study, considering the concurrent improvements in Yo-Yo IR2, RSA average scores, and lactate kinetics, *Rhodiola rosea*’s potential influence on energy availability and metabolic environment may represent one of the upstream factors contributing to this enhanced baseline state. Abidov et al. demonstrated in animal studies that *Rhodiola rosea* extract promotes skeletal muscle mitochondrial ATP synthesis or resynthesis while increasing ATP content, providing biological evidence for its potential energetics mechanism [66]. However, as mitochondrial function or ATP turnover were not directly measured, these mechanisms require further validation through subsequent research.

Overall, this study supports the notion that *Rhodiola rosea* may enhance performance maintenance capacity in football players during high-intensity intermittent scenarios by optimising energy metabolism and recovery kinetics. This improves neuromuscular output stability under fatigue conditions, thereby facilitating enhanced central information processing and decision-making performance. *Rhodiola rosea* supplementation was well tolerated, and no participant reported adverse events during the intervention.

This study retains certain limitations. The four-week intervention period remains insufficient to elucidate the adaptive trajectory and sustainability of longer-term supplementation. Furthermore, the absence of direct measurements of mitochondrial function, lactate transport, or neurophysiological indicators means current mechanistic explanations primarily rely on systemic physiological inferences. Additionally, the lack of plasma volume correction for haemoglobin and haematocrit results constrains definitive conclusions regarding haematopoietic or oxygen transport adaptations. Future research should employ larger samples and extended intervention periods, incorporating molecular, metabolic, and neurophysiological measurements to further validate *Rhodiola rosea*’s mechanisms of action during high-intensity interval training.

## 5. Conclusions

The present findings indicate that short-term *Rhodiola rosea* supplementation improves high-intensity intermittent performance under standardized fatigue conditions and, to some extent, preserves neuromuscular output and situational decision-making stability in soccer players, suggesting a potential role in attenuating fatigue-related performance decrements during match-related demands.

## Figures and Tables

**Table 1:** Basic Information of Subjects.

Group (*n* = 12)	Age (Years)	Height (cm)	Body Weight (kg)	BMI (kg·m^−2^)	Training Experience (Years)
RHO	20.3 ± 0.9	178.8 ± 5.5	72.4 ± 8.3	22.6 ± 1.2	8.1 ± 1.3
CTR	20.5 ± 0.8	179.6 ± 5	72.4 ± 7.5	22.4 ± 1.1	8.3 ± 1.2

**Table 2:** Daily Dietary Nutrient Composition of Participants.

Nutrient	CTR (*n* = 12)	RHO (*n* = 12)
Carbohydrates	453 ± 26 g	468 ± 25 g
Protein	145 ± 7 g	140 ± 8 g
Fat	60 ± 7 g	56 ± 6 g
Dietary Fiber	31 ± 5 g	34 ± 5 g

## Data Availability

The original contributions presented in this study are included in the article. Further inquiries can be directed to the corresponding authors.
